# High-Dose Dexamethasone Alters the Increase in Interleukin-16 Level in Adult Immune Thrombocytopenia

**DOI:** 10.3389/fimmu.2019.00451

**Published:** 2019-03-18

**Authors:** Xinru Wang, Lizhen Li, Yuanjian Wang, Xin Li, Qi Feng, Yu Hou, Chunhong Ma, Chengjiang Gao, Ming Hou, Jun Peng

**Affiliations:** ^1^Department of Haematology and Qilu Hospital, Shandong University, Jinan, China; ^2^Department of Haematology, Liaocheng People's Hospital, Liaocheng, China; ^3^Shandong Provincial Key Laboratory of Immunohematology, Qilu Hospital, Shandong University, Jinan, China; ^4^West China School of Medicine, Sichuan University, Jinan, China; ^5^Department of Immunology, Shandong University School of Medicine, Jinan, China

**Keywords:** adult immune thrombocytopenia, high-dose dexamethasone, Interleukin-16, caspase-3, Th1 polarization

## Abstract

Adult primary immune thrombocytopenia (ITP) is an autoimmune-mediated haemorrhagic disorder. Interleukin-16 (IL-16) can directly affect cellular or humoural immunity by mediating the cellular cross-talk among T cells, B cells and dendritic cells. Several studies have focused on IL-16 as an immunomodulatory cytokine that takes part in Th1 polarization in autoimmune diseases. In this study, we investigated IL-16 expression in the bone marrow supernatant and plasma of ITP patients and healthy controls. What's more, we detected IL-16 expression in ITP patients with the single-agent 4-day high-dose dexamethasone (HD-DXM) therapy. In patients with active ITP, bone marrow supernatant and plasma IL-16 levels increased (*P* < 0.05) compared with those of healthy controls. In the meantime, the mRNA expression in BMMCs (pro-IL-16, caspase-3) and PBMCs (pro-IL-16, caspase-3 and T-bet) of ITP patients was increased (*P* < 0.05) relative to those of healthy controls. In patients who responded to HD-DXM therapy, both plasma IL-16 levels and gene expression in PBMCs (pro-IL-16, caspase-3, and T-bet) were decreased (*P* < 0.05). In summary, the abnormal level of IL-16 plays important roles in the pathogenesis of ITP. Regulating Th1 polarization associated with IL-16 by HD-DXM therapy may provide a novel insight for immune modulation in ITP.

## Introduction

Adult primary immune thrombocytopenia (ITP) is an autoimmune-mediated haemorrhagic disorder in which platelets are destroyed by specific antibodies directed against platelet surface membrane glycoproteins (GPs) and prematurely cleared by macrophages in the reticuloendothelial system (1). The pathophysiology of ITP is characterized by excessive platelet destruction and a decrease in platelet production. The traditional mechanism of platelet destruction was GP-specific antibody mediated platelet clearance. Besides, skewed balance of T helper cell type 1 (Th1) to T helper cell type 2 (Th2) (2, 3), reduced and defective capability of regulatory T cells (4, 5), abnormal Th17 and Th22 cells (6), cytotoxic T lymphocyte (CTL)–mediated platelet destruction (7), and altered levels of cytokines including IL-11, IL-18, IL-27, and IL-35 were involved in the pathogenesis of ITP (8–11). However, there are several abnormalities contributing to the pathophysiology of ITP that remain to be explored.

Interleukin-16 (IL-16) is one of the first characterized cytokines with chemo-attractive activities for human T cells, which does not have any homology with other cytokines (12). The IL-16 gene is located on chromosome 15q26 in humans; it is translated into a 636-amino acid precursor, which was detected in both cytoplasm and nucleus (13). By activated caspase-3, pro-IL-16 is cleaved into a 121-amino acid carboxyl-terminal fragment (C-IL-16) and an amino-terminal prodomain (N-IL-16) in the cytoplasm, both of which have been found to be biologically active (14). The C-IL-16 is secreted into the plasma as a ligand for CD4/CD9 with chemo-attractant, growth factor, and differentiation factor capabilities on a variety of haematopoietic cell types that are involved in various specific autoimmune and inflammatory responses (15). The N-IL-16 is involved in the stabilization of p27Kip1 protein and retention of T lymphocytes in the G0/G1 cell cycle phase (16, 17).

IL-16 is secreted by several leukocyte subsets, including T cells (18, 19), eosinophils (20), mast cells (21, 22), monocytes (23), and dendritic cells (24). Moreover, B cells constitutively express pro-IL-16. IL-16 mediates the cross-talk between B cells and T cells via its chemotactic properties within lymph node follicles (25, 26). CD4, the common surface molecule, is known to be a receptor for IL-16 (27), while another surface molecule, CD9, has also been suggested as an alternate IL-16 receptor (28). Moreover, the effect of IL-16 on immune cells can be direct or indirect via the modulation of cytokines. For example, in addition to its role in regulating the recruitment and activation of CD4^+^ T cells to sites of inflammation (29), IL-16 stimulates the production of proinflammatory cytokines by monocytes, including interleukin 6, tumor necrosis factor-α, interleukin 1β, and interleukin 15, with related downstream biological effects (30).

An increasing number of studies have demonstrated IL-16 as an immunomodulatory cytokine that contributes to the regulation of recruitment and activation of CD4^+^ cell at sites with Th1 polarization in association with several autoimmune diseases, such as multiple sclerosis lesions (31) and autoimmune type 1 diabetes (32). The plasma level and gene expression of IL-16 in childhood ITP was previously studied by our team. Newly diagnosed ITP patients with a Th1-dominant immune phenotype exhibit a high level of plasma IL-16 (by ELISA) and IL-16 gene expression (by DNA microarray analysis) (33).

Adult ITP is an autoimmune disease with Th1 superiority(2, 3). Similarly, the quantitative and qualitative abnormalities of immunological cells and immune dysfunction in patients with ITP can be, at least in part, attributed to IL-16, suggesting the potential value of IL-16 in prognostic evaluation in ITP. The roles of pro-IL-16/mature IL-16 in ITP remain unknown. In this present study, the levels of IL-16 in the plasma and bone marrow supernatants and mRNA expression of pro-IL-16, caspase-3 and T-bet in the peripheral blood mononuclear cells (PBMCs) and bone marrow mononuclear cells (BMMCs) of patients with ITP were determined.

Treatment with high-dose dexamethasone (HD-DXM) as a single-agent for 4 days has been widely recognized as the first-line therapy for ITP patients in need of clinical management. Previous studies showed that HD-DXM could restore Th1/Th2 balance (34), expand regulatory cells including Tregs and MDSCs (35), suggesting the immunosuppressive function in recovery of ITP patients. However, the effects of HD-DXM on IL-16 expression remain unclear in ITP patients. Furthermore, we demonstrated changes in plasma levels of IL-16 and mRNA expression of pro-IL-16, caspase-3 and T-bet in PBMCs after HD-DXM, and these results may provide new insights into the mechanism for treatment of ITP with HD-DXM.

## Materials and Methods

### Patients and Controls

Adult primary ITP patients with active disease were enrolled between May 2015 and December 2016 at the Department of Hematology, Qilu Hospital, Shandong University, Jinan, China. Patients were diagnosed according to recent guidelines (36) including clinical histories, somatoscopy, complete blood count and smear examination of peripheral blood. Patients and controls with complications such as Graves' disease, diabetes, hypertension, cardiovascular diseases, nervous system disease, neurological diseases, pregnancy, active infection, or connective tissue diseases, such as systemic lupus erythaematous (SLE), were excluded. The study was permitted by the Medical Ethical Committee of Qilu Hospital, Shandong University. Informed consent was obtained from all patients and controls before enrollment in the study.

Bone marrow samples were obtained from 23 patients, and 20 were used for ELISA (5 men and 15 women; range 24–63 years, median age 45 years; platelet counts ranging from 0 to 23 × 10^9^/L, median count 9 × 10^9^/L) and 14 for RT-PCR (4 men and 10 women; range 26–62 years, median age 41 years; platelet counts ranging from 0 to 23 × 10^9^/L, median count 9 × 10^9^/L). Peripheral blood samples were obtained from 64 patients, and among them, 21 received single-agent HD-DXM therapy. The peripheral blood samples were obtained before HD-DXM therapy and 28 days after HD-DXM administration for ELISA and RT-PCR. Complete response (CR) was defined as a platelet count ≥ 100 × 10^9^/L and an absence of bleeding. Response (R) was defined as a platelet count ≥ 30 × 10^9^/L, with at least a 2-fold increase from baseline, and an absence of bleeding. No response (NR) was defined as a platelet count < 30 × 10^9^/L, less than a 2-fold increase from baseline, or bleeding (37). The main clinical characteristics of these patients are presented in Table 1. Among the peripheral blood samples, 52 were used for ELISA (18 men and 34 women; range 19–64 years, median age 42 years; platelet counts ranging from 1 to 26 × 10^9^/L, median count 10 × 10^9^/L) and 33 for RT-PCR (12 men and 20 women; range 19–69 years, median age 41 years; platelet counts ranging from 1 to 26 × 10^9^/L, median count 10 × 10^9^/L) and 33 for RT-PCR (12 men and 20 women; range 19–69 years, median age 41 years; platelet counts ranging from 1 to 26 × 10^9^/L, median count 10 × 10^9^/L).

**Table 1:** Clinical characteristics of active ITP patients treated with HD-DMX.

**Patient NO**.	**Course of disease (month)**	**Bleeding symptoms**	**Response**	**Platelet counts(× 10^**9**^)**	**Counts**
				**Before**	**After**
1	0(10d)	PT	CR	1	144
2	3	EC	R	20	92
3	0(3d)	PT, GH	CR	7	195
4	0(16d)	No	NR	23	20
5	5	PT, EP	CR	7	131
6	0(3 days)	PT, GH	CR	1	158
7	10	No	CR	27	135
8	9	No	NR	6	11
9	1	PT, EC	R	18	75
10	7.5	PT	CR	11	195
11	2	PT, EP	CR	8	141
12	1	PT, GH	CR	1	102
13	5	PT	CR	12	107
14	1	EC	CR	5	195
15	24	No	R	14	31
16	0.5	PT, GUH, GH	CR	7	203
17	12	PT, EC	R	10	44
18	4	EC, GH	CR	4	156
19	9	NO	NR	10	16
20	4.5	EC	CR	9	159
21	14	GUH	CR	17	174
Median	4			9	135
Male: 7	Median Age:51y (range 39–63)				
Female:14	Median Age:36y (range 19–64)				

The healthy adult control group consisted of 38 blood donors, and 26 of the samples were used for ELISA (10 men and 16 women; range 23–66 years, median age 41 years; platelet counts ranging from 105 to 263 × 10^9^/L, median count 161 × 10^9^/L) and 19 for RT-PCR (6 men and 13 women; range 23–68 years, median age 42 years; platelet counts ranging from 105 to 290 × 10^9^/L, median count 156 × 10^9^/L) In addition, there were 10 bone marrow donors, and 10 samples were for ELISA test (3 men and 7 women; range 24–63 years, median age 45 years; platelet counts ranging from 110 to 300 × 10^9^/L, median count of 172 × 10^9^/L) and 9 for RT-PCR (3 men and 6 women; range 24–63 years, median age 45 years; platelet counts ranging from 110 to 295 × 10^9^/L, median count of 172 × 10^9^/L).

### Sample Preparation

The peripheral blood or bone marrow samples were kept in heparin anticoagulant vacutainer tubes for no more than 2 h before isolation. Cell-free (platelet- and mononuclear cell-free) plasma or cell-free bone marrow supernatant samples were prepared using a standardized two-step separation method (700 g for 10 min and 1,300 g for 20 min) as described previously to avoid the affection from cell lysis then settled in −80°C until test (38, 39). PBMCs or BMMCs were isolated by density gradient centrifugation using Ficoll-Paque (Pharmacia Diagnostic, Uppsala, Sweden) and stored at −80°C until RNA isolation.

### Enzyme-Linked Immunosorbent Assays

Cell-free plasma IL-16 was measured using commercial Quantikine enzyme-linked immunosorbent assay (ELISA) kits (R&D systems, Minneapolis, MN, USA) according to the manufacturer's instructions. The detection range for IL-16 is 31.2–2,000 pg/mL (sensitivity is 13.4 pg/mL). The differences in bone marrow or plasma IL-16 levels between patients and healthy controls were compared using Mann-Whitney *U*-test. The plasma IL-16 levels before and after treatment were compared used paired *t*-test and Wilcoxon signed-rank test.

### RNA Isolation and Quantitative Real-Time Polymerase Chain Reaction Analysis

Total RNA was isolated using TRIzol reagent (Invitrogen, Carlsbad, CA, USA), and converted to cDNA using PrimeScriptTM RT Master Mix (Perfect Real Time; Takara, Japan) according to the manufacturer's instructions. The mRNA expression of IL-16, Caspase-3, T-bet and β-actin (endogenous control) was quantified using SYBR Green Real-time PCR Master Mix (Toyobo, Japan) on an ABI PRISM 7,500 Sequence Detection System (Applied Bio-system, Foster City, CA, USA). The primers for all mRNA assays were intron spanning. The sequences of the amplification primers for human pro-IL-16, Caspase-3, T-bet, and β-actin are listed in Table 2. Each sample was analyzed in triplicate. The PCR amplification was performed for 40 cycles after initial denaturation (95°C, 5 min) with the following parameters: denaturation at 95°C for 15 s, annealing at 60°C (pro-IL-16, Caspase-3, T-bet, and β-actin) for 15 s and extension at 72°C for 45 s, with temperature transition rates of 20°C/s. Fluorescence signals were acquired after extension at 72°C. ABI Sequence Detection System software version (PE Applied Biosystems, Warrington, UK) was used to measure the cycle number at which fluorescence emission crossed the automatically determined Ct value. Because all steps were performed with equal efficiencies, the relative mRNA expression level of the target gene in each patient was calculated using the comparative cycle time (Ct) method (40). Briefly, the Ct values of the targets were corrected by subtracting the Ct value of β-actin from these values, which generated the ΔCt value. From this ΔCt value, the relative expression level for each target gene compared to β-actin was calculated using the following formulation: Relative mRNA expression = 2^−−xCt^. Differences in mRNA expression between patients and controls were analyzed using the Mann-Whitney *U*-test. Differences in mRNA expression between pre-treatment and post-treatment samples were compared using Wilcoxon signed-rank test. The correlation between pro-IL-16, Caspase-3, and T-bet mRNA expression and plasma IL-16 level was determined using the linear regression test. Statistical analyses were performed at a 2-tailed significance level of 0.05.

**Table 2:** Primers and conditions for the qRT-PCR experiments performed in this study.

**Gene**	**Primer sequence(5^**′**^-3^**′**^)**	**Annealing temperature (^**°**^C)**	**Product (bp)**
IL-16	(F) 5′-ATGCCCGACCTCAACTCC-3′		
	(R) 5′-CTAGGAGTCTCCAGCAGC−3′	60	389
Caspase-3	(F)5′-GGGGATCGTTGTAGAAGTCTAACT-3′		
	(R)5′-GCATACAAGAAGTCGGCCTCCACT-3′	60	159
T-bet	(F)5′-CATTGCCGTGACTGCCTACC-3′		
	(R)5′-GATGCTGGTGTCAACAGATGTG-3′	60	121
β-actin	(F)5′- TTGCCGACAGGATGCAGAA−3′		
	(R)5′- GCCGATCCACACGGAGTACT−3′	60	101

## Results

### Response to HD-DXM Treatment

Out of the 21 patients, 18 (6 males and 12 females, median age 41 years, range 19–64 years) responded effectively to the HD-DXM therapy according to the standard definition (41). In these responders, platelet counts after treatment ranged from 31 to 203 × 10^9^/L, with a median count of 135 × 10^9^/L, as shown in Table 1. No hemorrhage or other complications were apparent after treatment.

### Concentration of Il-16 in the Bone Marrow Supernatant and Plasma of Patients With Active ITP and Controls

**Figure 1:**
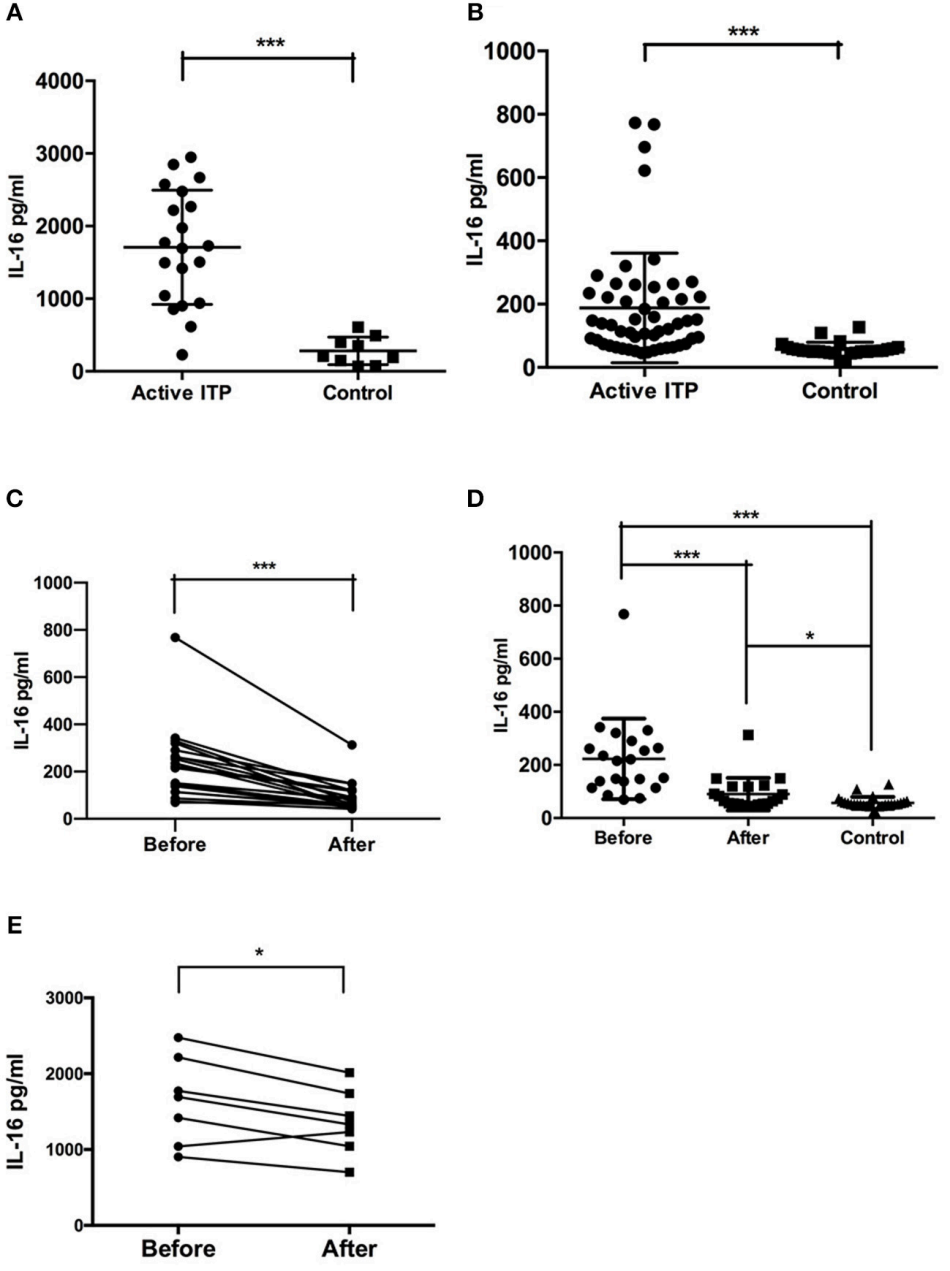
Bone marrow supernatant and plasma concentrations of IL-16 in active ITP and controls**. (A)** IL-16 levels in bone marrow in ITP patients and healthy controls were analyzed by ELISA. IL-16 levels were significantly higher in active ITP patients than healthy controls. **(B)** IL-16 levels in plasma in ITP patients and healthy controls were analyzed by ELISA. IL-16 levels were significantly higher in active ITP patients than healthy controls. ***P < 0.001. **(C)** Plasma concentration of IL-16 in active ITP before HD-DXM, after HD-DXM and controls. IL-16 levels in plasma in active ITP before HD-DXM and after HD-DXM were analyzed by ELISA. IL-16 levels were significantly higher in active ITP before HD-DXM than after HD-DXM treatment. **(D)** IL-16 levels in plasma in active ITP before HD-DXM, after HD-DXM and in controls were analyzed by ELISA. IL-16 levels were significantly higher in active ITP before HD-DXM than after HD-DXM treatment, and were significantly higher after HD-DXM treatment than in healthy controls. **(E)** IL-16 levels in bone marrow supernatants in active ITP before and after HD-DXM treatment were analyzed by ELISA. IL-16 levels were significantly decreased after HD-DXM treatment. ***P < 0.001; *P < 0.05.

The concentration of IL-16 in the bone marrow supernatants of ITP patients with active disease was significantly higher compared with that of the healthy controls (mean ± SEM: 1686 ± 195.6 (*n* = 19) vs. 287.1 ± 62.27 (*n* = 9), *P* = 0.0002, Mann-Whitney *U*-test) (Figure 1A).

The level of IL-16 in the plasma of ITP patients with active disease was significantly higher than that of healthy controls (mean ± SEM: 187.7 ± 24.06 (*n* = 52) vs. 57.14 ± 4.344 (*n* = 26), *P* < 0.0001, Mann-Whitney *U*-test) (Figure 1B).

Further experiments were performed to investigate the possible effects of HD-DXM on the activity of IL-16 in ITP patients. The level of IL-16 in the plasma of ITP patients was detected before and 7 days after single-agent HD-DMX therapy for 4 days. The post-treatment plasma IL-16 level decreased significantly relative to that of pre-treatment level (mean ± SEM: 222.9 ± 33.09 (*n* = 21) vs. 90.17 ± 13.36 (*n* = 21), *P* = 0.0003, paired *t*-test) (Figure 1C). The level of IL-16 in the plasma of ITP patients before (*P* < 0.0001) and after (*P* = 0.0143) HD-DXM treatment was significantly higher than that of healthy controls, 57.14 ± 4.344 (*n* = 26), Mann-Whitney *U*-Test) (Figure 1D). The level of IL-16 in bone marrow supernatants was also downregulated after 4-day HD-DXM therapy (mean ± SEM: 1646.16 ± 578.11 (*n* = 7) vs. 1358.07 ± 433.79 (*n* = 7), *P* = 0.016, paired *t*-test) (Figure 1E).There was no correlation between IL-16 levels in the bone marrow supernatants or plasma and platelet count (data not shown).

### mRNA Expression Levels of Pro-IL-16, Caspase-3 and T-Bet in ITP Patients and Controls

To elucidate the elevated plasma concentration of IL-16 in patients with active ITP, we determined the mRNA expression of pro-IL-16, caspase-3, and T-bet. Previous studies have demonstrated that high mRNA expression of caspase-3 is necessary for the processing and activation of pro-IL-16 (14). Therefore, we determined caspase-3 mRNA expression in our study. Several studies have demonstrated that IL-16 could skew immune responses toward a Th1 response (42, 43). Therefore, T-bet mRNA expression was also analyzed in our study to further evaluate the relationship between IL-16 and Th1 deviation in ITP Patients. Relative expression of mRNAs was calculated using 2^−ΔCT^, and then the means were compared using *t*-test or Mann-Whitney *U*-test depending on the variance.

**Figure 2:**
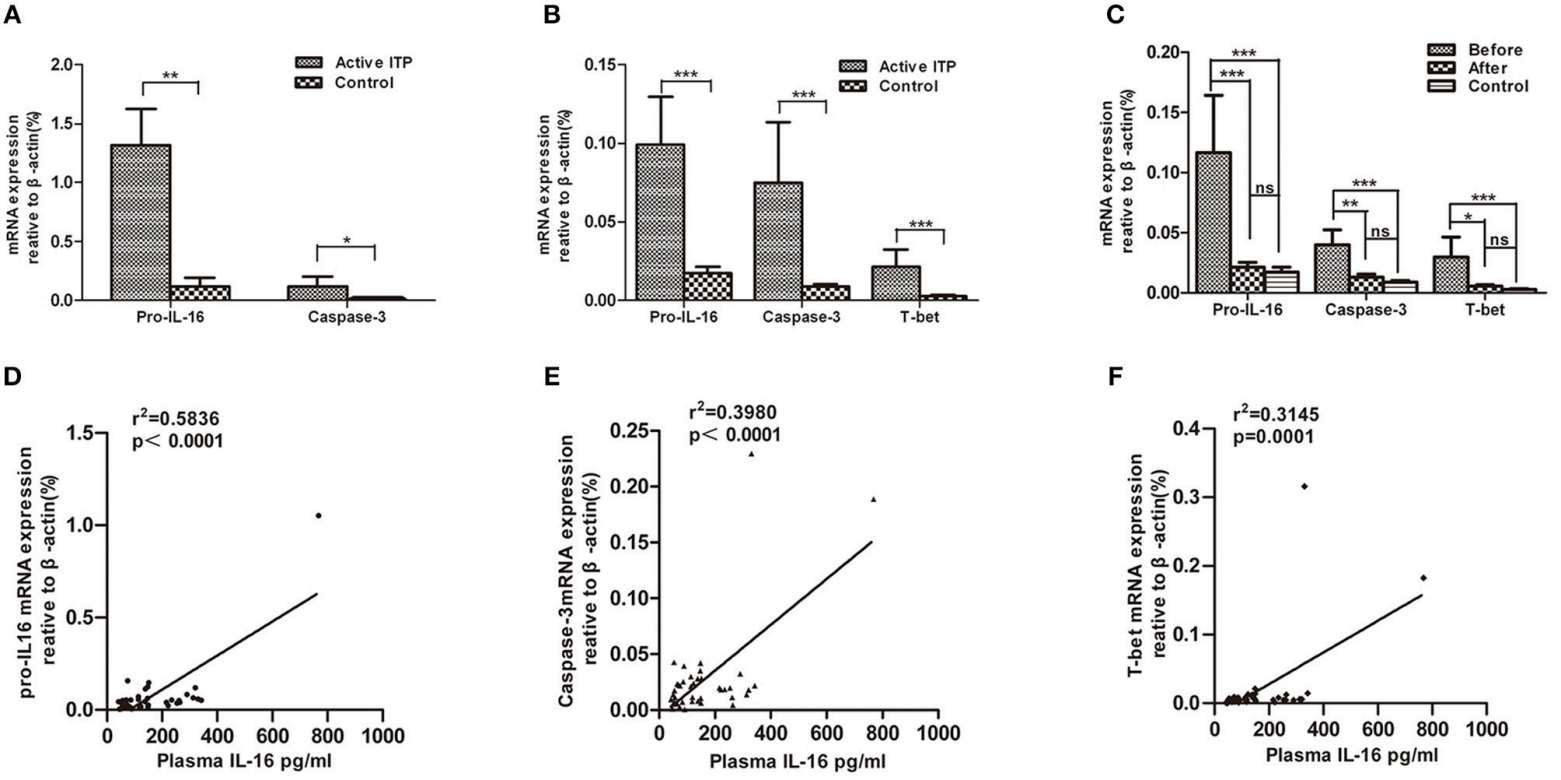
Relative mRNA expression of pro-IL16, caspase-3 and T-bet in ITP patients with active disease and healthy controls. mRNA expression in bone marrow mononuclear cells (BMMCs) and peripheral blood mononuclear cells (PBMCs) from ITP patients and healthy controls were quantified by real-time PCR. Furthermore, correlations between plasma IL-16 and mRNA expression levels of pro-IL16, caspase-3 and T-bet in active ITP patients were calculated. **(A)** Relative mRNA expression of pro-IL16, caspase-3 in ITP patients with active disease and healthy controls in BMMCs; ***P < 0.001; ** *P* < 0.01; * *P* < 0.05. **(B)** Relative mRNA expression of pro-IL16, caspase-3 and T-bet in PBMCs in ITP patients with active disease and healthy controls. *** *P* < 0.001; ** *P* < 0.01; * *P* < 0.05. **(C)** mRNA expression of pro-IL16, caspase-3 and T-bet in active ITP before HD-DXM, after HD-DXM and controls. *** *P* < 0.001; ** *P* < 0.01; * *P* < 0.05. **(D)** Correlation between plasma and mRNA expression levels of IL-16 in active ITP patients. Plasma IL-16 levels and pro-IL-16 mRNA expression in PBMCs were determined by ELISA and real-time PCR. There was a positive correlation between the factors (*r*^2^ = 0.5846, *P* < 0.0001; Pearson correlation analysis). **(E)** Correlation between plasma IL-16 levels and caspase-3 mRNA expression in active ITP patients. Plasma IL-16 levels and caspase-3 mRNA expression in PBMCs were determined by ELISA and real-time PCR. There was a positive correlation between the factors (*r*^2^ = 0.3980, *P* < 0.0001; Pearson correlation analysis). **(F)** Correlation between plasma IL-16 levels and T-bet mRNA expression levels in active ITP patients. Plasma IL-16 levels and T-bet mRNA expression in PBMCs were determined by ELISA and real-time PCR. There was a positive correlation between the factors (*r*^2^ = 0.3145, *P* < 0.0001; Pearson correlation analysis).

In accordance with the concentration of IL-16 in the bone marrow supernatants, the pro-IL-16 mRNA expression of BMMCs in patients with active ITP was significantly up-regulated compared with that of healthy controls (1.317 ± 0.3074 (*n* = 14) vs. 0.1182 ± 0.7577 (*n* = 9), *P* = 0.0012); caspase-3 mRNA were significantly higher in ITP patients compared with that in healthy controls (0.1194 ± 0.0797 (*n* = 14) vs. 0.006919 ± 0.001468 (*n* = 9), *P* = 0.0298) (Figure 2A).

Consistent with the plasma IL-16 level, the relative amount of mRNA of pro-IL-16 and caspase-3 of PBMCs in patients with active ITP was 0.09917 ± 0.03048 (*n* = 33, *P* < 0.0001) and 0.07495 ± 0.03843 (*n* = 33, *P* < 0.0001) relative to that in healthy controls (0.02230 ± 0.006397 and 0.009746 ± 0.001849, respectively; *n* = 19). However, both pro-caspase 3 and active caspase 3 protein levels have no significant difference in ITP patients compared with healthy control (Supplemental Figure). Our previous study has shown that T-bet mRNA expression was statistical higher in patients with active ITP than that in normal controls (44), which was in accordance with our present results where T-bet mRNA expression was significantly higher in patients with active ITP than that in controls (0.02148 ± 0.01098% (*n* = 33) vs. 0.002748 ± 0.0005696 (*n* = 19), *P* < 0.0001) (Figure 2B). Pro-IL-16, Caspase-3 and T-bet mRNA expression after 4 days of treatment with HD-DMX as a single agent compared with that before treatment was significantly decreased, although the corresponding values were not statistically higher than those of healthy controls (Figure 2C).

### Correlation of Plasma IL-16 Level With Pro-IL-16, Caspase-3 and T-Bet mRNA Levels in ITP Patients

The correlation between the plasma IL-16 concentration and pro-IL-16, caspase-3 and T-bet mRNA levels was analyzed in ITP patients. The results demonstrated that the mRNA levels of the three detected factors were positively correlated with plasma IL-16 concentration (*r*^2^ = 0.5846, *P* < 0.0001, *r*^2^ = 0.3980, *P* < 0.0001, and *r*^2^ = 0.3145, *P* = 0.0001 for pro-IL-16, caspase-3, and T-bet, respectively; linear regression; Figures 2D–F.

Taken together, in this study we demonstrated that the IL-16 levels in the bone marrow supernatants and plasma of adult ITP patients with active disease were significantly higher than those in healthy controls. The mRNA expression of IL-16, caspase-3 and T-bet in PBMCs and BMMCs were statistically higher than the corresponding mRNA expression in healthy controls. Single-agent HD-DMX treatment for 4 days decreased the plasma IL-16 level and downregulated mRNA expression of IL-16, caspase-3, and T-bet in ITP patients PBMCs. Furthermore, the plasma IL-16 levels were positively correlated with pro-IL-16, caspase-3, and T-bet mRNA expression.

## Discussion

Our results provide evidence that IL-16 contributes to the development of ITP. The mechanism by which IL-16 promotes ITP requires a considerable amount of work to be fully understood. IL-16 is a special cytokine with two isoforms, namely, N-IL-16 and C-IL-16, and both of these isoforms have been shown to have modulatory functions for growth and activation of immune cell (14). Elevation of C-IL-16 levels at sites of inflammation may contribute to Th1 advantage, in consistent with the previously reported role of this protein in other Th1-associated immune diseases (42, 45). Lynch,et al. demonstrated that IL-16 preferentially induces Th1 cell migration with help from CCR5. Th1 subset specificity is attributable to an increase in IL-16 binding (46). Recently, our team had demonstrated that in ITP patients, CCR5 expression in PBMCs of patients with active ITP was significantly higher than in PBMCs of healthy controls. These results may explain how C-IL-16 alters the Th1/Th2 balance in ITP patients (47). N-IL-16 is located both in the cytolymph, as a substrate for mature IL-16 following caspase-3 dissociation, and in the cyteblast where it functions to weaken the cleavage of p27Kip1, a pivotal cell-cycle molecules (48). Meanwhile,the IL-16 expression was significantly down-regulated by HD-DXM therapy. We suppose the role of decreased IL-16 in the pathogenesis of ITP might including: reducing the production of proinflammatory cytokines by monocytes (30); decreasing the generation of GP-specific antibody and correcting the imbalance of Th1/Th2 differentiation (31),(32).

Increasing evidence has shown that an abnormally strong Th1 response plays a central role in the pathogenesis of chronic ITP (2, 3). T-bet is a T-box family transcription factor whose expression is primarily limited to the immune system. It is present in early developing Th1 cells but is absent in developing Th2 cells (49). We also found high levels of T-bet mRNA expression of ITP patients and further determined that plasma IL-16 levels positively correlated with Th1 levels in these patients.

ITP is an organ-specific autoimmune disorder; complex interactions among antigen-presenting cells, T cells and B cells are pivotal to its pathogenesis. In particular, B cells not only produce immunoglobulin but also play an important immunoregulatory role in the pathophysiology of ITP (41). A study reported that IL-16 is necessary for B lymphocytes to attract dendritic cells and Th1 cells (25). A novel monoclonal anti-IL-16 antibody called 14.1, that combines with IL-16 and induces a conformational change in the IL-16 PDZ domain has been recently reported (50). When incubated with CD4^+^ cells, the 14.1 antibody was shown to reduce the Th1-type inflammatory response. In our future study, we will use an *in vitro* study system to test and verify if the anti-IL-16 antibody can be used to treat adult ITP.

Our data suggest that IL-16 plays an important role in the pathogenesis of ITP by polarization of Th1. Further, by modulating the abnormal IL-16 level associated with the Th1 imbalance via treatment with pulsed HD-DXM provided us with new insights into the immune regulatory mechanisms for the treatment of ITP.

## Ethics Statement

This study was carried out in accordance with the recommendations of Medical Ethical Committee of Qilu Hospital, Shandong University with written informed consent from all subjects. All subjects gave written informed consent in accordance with the Declaration of Helsinki. The protocol was approved by the Medical Ethical Committee of Qilu Hospital, Shandong University.

## Author Contributions

JP and XW designed research, analyzed data, and wrote the paper. LL, YW, and XL performed research, analyzed data. QF and YH performed research and wrote the paper. CM and CG evaluated the data and corrected the paper. MH reviewed the manuscript. All authors read and approved the final manuscript.

### Conflict of Interest Statement

The authors declare that the research was conducted in the absence of any commercial or financial relationships that could be construed as a potential conflict of interest.
