# Supplementary material for: High-Dose Dexamethasone Alters the Increase in Interleukin-16 Level in Adult Immune Thrombocytopenia
Source: Front Immunol. 2019 Mar 18;10:451. doi: 10.3389/fimmu.2019.00451 (PMC6431608; doi:10.3389/fimmu.2019.00451)

Supplemental Data

Supplemental Figure: Both pro-caspase 3 and active caspase 3 protein levels have no significant difference in ITP patients compared with healthy control.

Peripheral blood mononuclear cell (PBMC) was obtained from 5 active ITP patients and 5 healthy controls were enrolled randomly and lysed in NP-40 lysis buffer (Beyotime, Nantong, China). Total proteins were fractionated by sodium dodecyl sulfate polyacrylamide gel electrophoresis (SDS-PAGE) and transferred to nitrocellulose membrane. The membranes were then incubated with rabbit polyclonal anti-caspase 3 antibody (Cell Signaling Technology, Danvers, MA, USA) or anti-β actin antibody (Abcam, Cambridge, MA, USA), followed by incubation with secondary goat anti-rabbit IgG H&L conjugated with horse radish peroxidase HRP (Abcam).

Our results showed that slightly higher expression of pro-caspase-3 (32KD) and lower expression of active caspase-3 (20KD) in active ITP patients compared with healthy controls, but no significant difference was observed. Caspase 3 is associated with induction of apoptosis. Pro-caspase-3 (32KD) was activated by cleavage to generate a subunit of 20KD and play a vital role in cell apoptosis. In our study, increased mRNA expression of caspase-3 was observed, which was positively correlated with plasma IL-16 in ITP patients. Previous study has demonstrated that high mRNA expression of caspase-3 is necessary for the processing and activation of pro-IL-16. However, their study also showed that apoptosis is not a requirement for IL-16 release, implying caspase-3 might be utilized in a nonapoptotic pathway independent on its activation1. In our study, low active caspase-3 expression in ITP patients also indicate that caspase-3 might exert its effect on IL-16 through mechanisms other than apoptosis.

Supplemental Figure

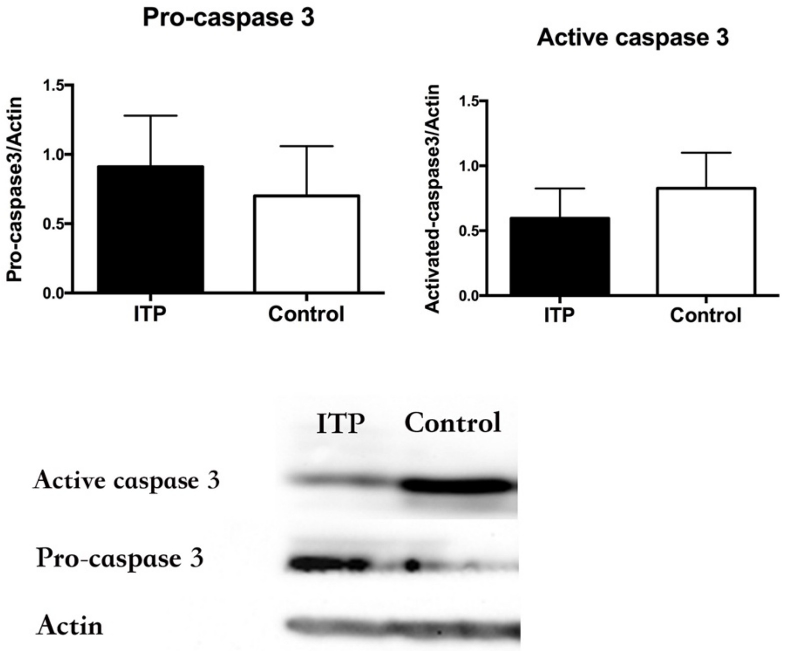

Supplement: Supplementary file 1 [file Data_Sheet_1.PDF]
